# Postprandial changes in cardiometabolic disease risk in young Chinese men following isocaloric high or low protein diets, stratified by either high or low meal frequency - a randomized controlled crossover trial

**DOI:** 10.1186/s12937-016-0141-5

**Published:** 2016-03-15

**Authors:** Alexander Mok, Sumanto Haldar, Jetty Chung-Yung Lee, Melvin Khee-Shing Leow, Christiani Jeyakumar Henry

**Affiliations:** 1 Clinical Nutrition Research Centre, Singapore Institute for Clinical Sciences, 14 Medical Drive, #07-02, Singapore, 117599 Singapore; 2 Singapore Institute for Clinical Sciences, Agency for Science, Technology and Research (A*STAR), 30 Medical Drive, Singapore, 117609 Singapore; 3 Department of Biochemistry, National University of Singapore, 8 Medical Drive, Singapore, 117596 Singapore; 4 School of Biological Sciences, Kadoorie Biological Sciences Building, The University of Hong Kong, Pok Fu Lam Road, Hong Kong, SAR China; 5 Division of Medicine, Department of Endocrinology, Tan Tock Seng Hospital, 11, Jalan Tang Tock Seng, Singapore, 308433 Singapore

**Keywords:** High protein, Meal frequency, Cardiometabolic disease risk

## Abstract

**Background:**

Cardio-Metabolic Disease (CMD) is the leading cause of death globally and particularly in Asia. Postprandial elevation of glycaemia, insulinaemia, triglyceridaemia are associated with an increased risk of CMD. While studies have shown that higher protein intake or increased meal frequency may benefit postprandial metabolism, their combined effect has rarely been investigated using composite mixed meals. We therefore examined the combined effects of increasing meal frequency (2-large vs 6-smaller meals), with high or low-protein (40 % vs 10 % energy from protein respectively) isocaloric mixed meals on a range of postprandial CMD risk markers.

**Methods:**

In a randomized crossover study, 10 healthy Chinese males (Age: 29 ± 7 years; BMI: 21.9 ± 1.7 kg/m^2^) underwent 4 dietary treatments: CON-2 (2 large Low-Protein meals), CON-6 (6 Small Low-Protein meals), PRO-2 (2 Large High-Protein meals) and PRO-6 (6 Small High-Protein meals). Subjects wore a continuous glucose monitor (CGM) and venous blood samples were obtained at baseline and at regular intervals for 8.5 h to monitor postprandial changes in glucose, insulin, triglycerides and high sensitivity C-reactive protein (hsCRP). Blood pressure was measured at regular intervals pre- and post- meal consumption. Urine was collected to measure excretion of creatinine and F_2_-isoprostanes and its metabolites over the 8.5 h postprandial period.

**Results:**

The high-protein meals, irrespective of meal frequency were beneficial for glycaemic health since glucose incremental area under the curve (iAUC) for PRO-2 (185 ± 166 mmol.min.L^−1^) and PRO-6 (214 ± 188 mmol.min.L^−1^) were 66 and 60 % lower respectively (both *p* < 0.05), compared with CON-2 (536 ± 290 mmol.min.L^−1^). The iAUC for insulin was the lowest for PRO-6 (13.7 ± 7.1 U.min.L^−1^) as compared with CON-2 (28.4 ± 15.6 U.min.L^−^1), *p* < 0.001. There were no significant differences in postprandial responses in other measurements between the dietary treatments.

**Conclusions:**

The consumption of composite meals with higher protein content, irrespective of meal frequency appears to be beneficial for postprandial glycemic and insulinemic responses in young, healthy Chinese males. Implications of this study may be useful in the Asian context where the consumption of high glycemic index, carbohydrate meals is prevalent.

**Trial registration:**

NCT02529228.

## Background

Increases in postprandial glucose, insulin and lipids have been linked to increased cardiometabolic disease (CMD) risk [1–3], even though the majority of epidemiological and intervention trials have relied on measuring these parameters in the fasted state. Given that most individuals spend majority of their waking hours in the postprandial state, eating and digesting food, fasting measurements alone may not, in its entirety, represent the metabolic processes indicative of CMD risk [4]. Moreover, Asians are at a greater risk of CMD than other populations such as the Caucasians [5] and several studies reported Asians having higher levels of fasting and postprandial glucose and insulin responses [6, 7], higher levels of HbA_1C_ [8] and increased type 2 diabetes risk compared with Caucasians [9, 10]. Asians also have a lower metabolic capacity than Caucasians to handle an excess dietary load [11]. Therefore alterations in meal composition and patterns are warranted in order to improve postprandial metabolism amongst Asians.

Increasing meal frequency has long been suggested by several authors to improve metabolic health and weight control [12–14]. It has been shown that eating smaller and frequent meals seems to better maintain blood glucose, blood lipid and incretin hormone concentrations, with lower variations throughout the day [14, 15]. However, the evidence thus far relating to the effects of meal frequency alone on glycaemic response remain equivocal with some studies showing a greater postprandial glucose responses with increased meal frequency [16, 17], while other studies reporting lower blood glucose responses with higher meal frequencies in non-diabetics [14] as well in diabetics [18, 19]. There have also been studies reporting no difference in postprandial glycaemia with increased meal frequency [20, 21]. However, the compositions of the meals were rather heterogenous between these studies and this may explain the disparate findings observed between studies investigating the effect of meal frequency alone.

It is widely known that Asian diets are particularly rich in refined carbohydrates which may exacerbate the risk of type 2 diabetes and CVD [22]. A recent review has highlighted that the prevalence of diabetes worldwide also parallels the increases in carbohydrate intake over the same period [23]. Therefore, modulating dietary carbohydrate content and composition has been suggested to be an effective way of decreasing glycaemia [24, 25]. Recent evidence suggests that consuming higher amounts of protein can lead to a lower CMD risk [26–29]. In particular, greater protein to carbohydrate ratios in the diet have been shown result in better glycaemic control [29–32].

Therefore the primary aim of this study was to investigate whether altering protein composition with or without increasing meal frequency can improve postprandial glucose and/or insulin response in young, healthy, Chinese males. Since postprandial glycemic control is also associated with changes in triglyceride, hs-CRP and markers of oxidative stress [2], as secondary outcomes, we have also measured postprandial changes in these markers in plasma as a result of the acute dietary modulations.

## Methods

### Participants

This randomized control trial was conducted at the Clinical Nutrition Research Centre (CNRC) within the Singapore Institute of Clinical Sciences (SICS), Agency of Science Technology and Research (A*STAR), Singapore, between October 2014 to July 2015. The study received ethical approval from the Domain Specific Review Board of the National Healthcare Group in Singapore (NHG DSRB Reference No. 2014/01054). All subjects gave their written informed consent before participating in the study. Research procedures and trial protocols were followed in accordance to good clinical practice (GCP) guidelines and with the ethical standards in concordance to the Declaration of Helsinki, 1983. This trial is registered within clinicaltrials.gov under trial registration no. NCT02529228.

Healthy Chinese males between the ages of 21–40 years were recruited for this trial. The exclusion criteria for the study were: 1) smokers, 2) BMI > 25 kg/m^2^; 3) fasting blood glucose > 5.5 mmol/l; 4) blood pressure > 130/90 mmHg; 5) body weight change > 3 kg within the previous 2 months; 6) having taken part in any dietary intervention trials or having followed a special dietary habit (e.g., vegetarian, Atkins, weight reducing diets) within 3 months of study participation; 7) suffering from any endocrine (thyroid dysfunction) or metabolic (dyslipidemia, diabetes, metabolic syndrome) disorders; and 8) alcohol use > 4 days per week. Volunteers who met the initial inclusion/exclusion criteria at screening were invited to take part in the study. A total of 10 volunteers were recruited and there were no withdrawals from the study. Participant characteristics are summarized in Table 1.

**Table 1:** Participant’s characteristics

		Range	
Characteristic	Mean (SD)	Min	Max
Age (Years)	28.8 (6.6)	22	40
Height (m)	1.76 (0.04)	1.71	1.84
Body Mass (kg)	68.2 (7.8)	58.1	83
Body Mass Index (kg/m^2^)	21.9 (1.7)	19.5	24.5
Umbilical Waist Circumference (cm)	80.5 (7.9)	70	92
Body Fat %	17.0 (4.7)	8.9	22.6
Systolic Blood Pressure (mmHg)	120.4 (5.7)	113	130
Diastolic Blood Pressure (mmHg)	76.1 (6.4)	65	85

### Design

The study was a randomized, crossover trial with four acute dietary conditions, comparing postprandial effects of varying two parameters of: 1) meal frequency and 2) dietary protein composition, in a 2-by-2 factorial design. Participants were studied under 4 separate 1-day isocaloric nutritional interventions: CON-2 (2 large Low-Protein meals :10 % Protein, 57 % Carbohydrate, 38 % Fat); CON-6 (6 Small Low-Protein meals with identical nutrient composition as CON-2); PRO-2 (2 Large High-Protein meals :38 % Protein, 30 % Carbohydrate, 32 % Fat); PRO-6 (6 Small High-Protein meals with identical nutrient composition as PRO-2). The order of the sessions was randomized with a washout period of at least 3 days between interventions. Participants refrained from alcohol consumption and vigorous exercise 48-h before each trial session. The evening before each session, participants came to the clinical suite for the insertion of a continuous glucose monitor (CGM) (Medtronics, USA) under the abdominal subcutaneous adipose tissue by a trained researcher and also for the consumption of a standardized evening meal. Participants fasted for 12-h following their evening meal up to the start of each session. The CGM measured interstitial glucose levels in 5 min intervals for the entire duration of the sessions.

### Study protocol

**Fig. 1:**
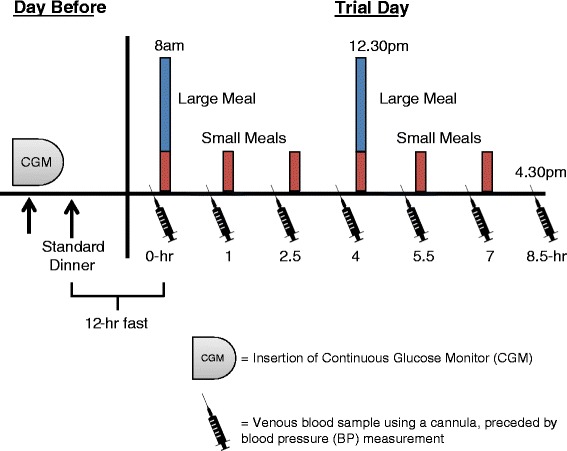
Study Protocol: For each dietary condition, participants came to the clinical trial facilities on 2 consecutive days. The day before the main trial day was for the insertion of a continuous glucose monitor and the consumption of a standardized evening meal. On the main trial day, BP and venous blood samples were collected at regular intervals, as shown, for up to 8.5 h following the first meal of each dietary condition

The schematic of the study protocol is shown in Fig. 1. On the morning of each test day, the participants arrived in a fasting state. Throughout each test session, lasting 8.5 h during the day (0800–1630 h), participants stayed within a clinical suite and predominantly remained in a seated posture doing passive work (i.e., watching TV, using computers or reading quietly). The temperature of the suite was kept under thermo-neutral conditions (air-conditioned to keep within 22–24 degree Celsius ((°C)) throughout the entire study period. At the start of each test day, an indwelling venous cannula was inserted into the participant’s antecubital vein by a trained nurse for the collection of baseline and subsequent regular blood sampling. This cannula was kept patent by flushing with 3 ml non-heparinized saline. After the fasting baseline blood sample was taken, participants consumed high or low protein isocaloric mixed meals in either a 2-meal or 6-meal frequency pattern over the subsequent 8.5 h period. The total energy content of the meals (approximately 2000 kcals) were designed to provide a small caloric surplus based on two-thirds (from breakfast and lunch) of the daily energy requirements for an average sedentary male (Singapore National Nutrition Survey, 2010) [33]. This would reflect the typical overconsumption of calories in modern day society. For the 2-meal trials (CON-2 and PRO-2), test meals consisted of 1 large breakfast meal and 1 large lunch meal, whereas for the 6-meal trials (CON-6 and PRO-6), the breakfast and lunch were each divided into 3 equal, isocaloricl portions. Meal times were scheduled at equidistant time points (Fig. 1); that is, during the 2-meal sessions, meals were provided at 4.5-h intervals (0800 and 1230-h), and during the 6-meal sessions, meals were served at 1.5-h intervals (0800, 0930, 1100, 1230, 1400, and 1530). Two of the meals during the 6-meal trials were provided at the same time as the meals during the 2-meal conditions (i.e., 0800 and 1230). Postprandial blood samples and blood pressure measurements were taken at 1 to 1.5-h intervals. Participants also collected their entire urine passed during the 8.5-h postprandial period for the analyses of urinary F_2_-isoprostanes, its metabolites and creatinine excretion.

The energy and the macronutrient compositions, along with the food amounts and constituents and the estimated glycemic indices (GIs) of the test meals are detailed in Table 2. The estimated GIs of each composite meal were calculated according to the method by Hatonen KA et al. [34]. The GI of individual foods used for the study were derived using published GI data of foods same or similar foods [35, 36]. Based on these calculations for the low-protein CON-2/CON-6 meals, the overall GI was estimated to be 62, whereas, for the high-protein PRO-2/PRO-6 meals, the overall GI was estimated to be 31. Volunteers were required to consume all of their meals as provided and no other food or drinks were permitted. Researcher surveillance of study participants during meal times and collection of empty food containers ensured dietary compliance. Participants were asked to consume each meal within 15-mins of serving. There were no complaints of any gastrointestinal or other discomforts after consuming the test meals. Plain drinking water was provided *ad libitum* during first test day and this volume was consumed for the remaining test sessions. All blood samples were collected in K-EDTA blood vacutainer tubes (Becton Dickinson Diagnostics, USA) and were centrifuged at 4500 rpm for 10 mins at 4 °C. The plasma so obtained was immediately stored at −80 °C until batch analysis of separate analytes at the completion of the clinical trial. Subcutaneous interstitial fluid glucose readings were obtained every 5-min for the entire 8.5 h postprandial period using the CGM. Appropriate calibrations with capillary blood glucose were also made for this purpose. Plasma samples were assayed for insulin, triacylglycerol (TAG), and high-sensitivity C-reactive protein (hs-CRP) using an immunochemistry analyzer COBAS e411 (Roche, HITACHI, USA). Urinary F_2_-Isoprostanes (5-F_2t_-isoprostane and 15-F_2t_-isoprostane) and its metabolites (2,3-dinor-15-F_2t_-isoprostane and 2,3-dinor-5,6-dihydro-15-F_2t_-isoprostane) were measured using a LC-MS/MS method [37, 38] and creatinine was measured using the COBAS e411 analyser (Roche, Hitachi, USA).

**Table 2:** Nutritional information of test-meals

CON-2, CON-6 dietary conditions			
Meal Constituents: Breakfast – White Bread (171 g), Margarine (30 g), Malted Cocoa Drink (375 g)			
Lunch – Chicken Soup Noodles (225 g), Cashew Nuts (30 g)			
	Breakfast	Lunch	Total
Energy (kcal)	889	1127	2016
Protein (g)	23 g (10 %)	26 g (9 %)	49 (10 %)
Carbohydrate (g)	130 g (57 %)	134 g (42 %)	264 (52 %)
Fat (g)	32 g (33 %)	53 g (49 %)	85 (38 %)
Estimated Glycaemic Index	73.4	50.3	61.7
PRO-2, PRO-6 dietary conditions			
Meal Constituents: Breakfast – Milk (300 g), Ice-Cream (150 g), Whey powder (91.2 g), Cashew Nuts (30 g), Lunch – Braised Chicken (330 g), Prawn Dumplings (120 g), Malted Cocoa Drink (375 g)			
	Breakfast	Lunch	Total
Energy (kcal)	910	998	1908
Protein (g)	89 g (39 %)	90 g (36 %)	179 (38 %)
Carbohydrate (g)	66 g (30 %)	75 g (31 %)	141 (30 %)
Fat (g)	31 g (31 %)	37 (33 %)	68 (32 %)
Estimated Glycaemic Index	37.1	24.9	30.6

Macronutrient information obtained from nutrition labels on constituent foods

## Statistical analyses

The initial sample size was calculated to detect a difference between dietary conditions in the primary outcome measures, being both postprandial glycemia and insulinemia, using primary data obtained from a similar dietary intervention by Holmstrup M et al. [17]. A sample size of 9 was estimated to detect a difference with a 80 % power and a significance (α) level set at 0.05 using a crossover design. The incremental area under the curve (iAUC) for postprandial CGMS glucose, plasma insulin, triglycerides and hs-CRP as well as blood pressure (BP) were calculated using the trapezoidal method [39] and compared between dietary treatments using a one-way ANOVA. The concentrations of glucose, insulin, TAG, hsCRP and BP were compared between treatments and over time using two-way repeated measures ANOVA. Where appropriate, Bonferroni post hoc comparisons were used. Data are presented as means (standard deviations, SD) unless otherwise defined. Significance was set at *p* < 0.05. Statistical analyses were carried out using Statistical Package for the Social Sciences statistical software, version 23.0 (IBM Inc.).

## Results

**Fig. 2:**
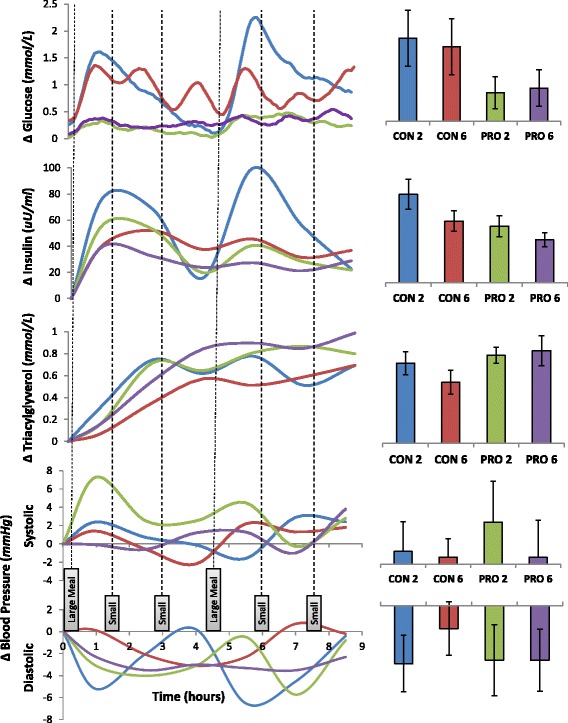
Postprandial responses over time (*left*); Incremental Areas Under the Curve (*Right*). Error bars are standard error of the mean (SEM)

Figure 2 displays the 8.5 h postprandial response, depicted as mean incremental areas under the curve (iAUCs) for glucose, insulin, triacylglycerol (TAG), systolic and diastolic blood pressure for each of the 4 dietary conditions. One-way repeated measures ANOVA for the mean iAUCs showed significant differences overall, between the dietary treatments for glucose (*P* = .003, η^2^ = .399), insulin *(P* = .001, η^2^ = .433) and TAG (*P* = .026, η^2^ = .287). Table 3 summarizes the pairwise comparisons of the means between dietary conditions across time as well as the differences in the iAUCs for each dietary treatment and their respective effect size (*d*) as compared with CON-2.

**Table 3:** Postprandial responses across time

Time (h)	0	1	2.5	4	5.5	7	8.5	iAUC	Overall p (Diet)	*D*
Glucose (mmol/L)										
CON-2	4.7 ± 0.6	6.1 ± 1.1^a^	5.4 ± 0.6^a^	4.8 ± 0.8^a^	6.8 ± 1.0^a^	5.7 ± 0.5	5.4 ± 0.8	536 ± 290	.003	
CON-6	5.0 ± 0.8	6.2 ± 1.1	5.9 ± 0.5^b^	5.8 ± 0.6^b^	6.1 ± 0.5^a^	5.7 ± 0.7^a^	6.1 ± 1.1	480 ± 292		- 0.19
PRO-2	4.8 ± 0.4	5.2 ± 0.4^b^	5.0 ± 0.4^a^	4.9 ± 0.4^a^	5.2 ± 0.6^b^	5.2 ± 0.7	5.0 ± 0.8	185 ± 166		- 1.21
PRO-6	4.7 ± 0.4	5.2 ± 0.4	5.0 ± 0.4^a^	5.1 ± 0.4^a^	5.1 ± 0.5^b^	5.1 ± 0.6^b^	5.2 ± 0.7	214 ± 188		- 1.11
Insulin (μU/ml)										
CON-2	6.1 ± 3.3	84.3 ± 45.3^a^	74.0 ± 43.6^a^	21.6 ± 16.8	105.6 ± 61.0^a^	62.0 ± 46.4	28.6 ± 17.9	28.4 ± 15.6 ^a^	.001	
CON-6	8.1 ± 5.0	49.7 ± 19.5	60.1 ± 31.9	45.6 ± 29.2^a^	53.7 ± 33.2	39.5 ± 22.0	44.8 ± 24.9	19.8 ± 10.3		- 0.57
PRO-2	6.0 ± 4.2	63.3 ± 41.2	58.7 ± 36.2	25.6 ± 18.5^b^	46.6 ± 22.5	34.4 ± 24.9	27.8 ± 25.9	18.1 ± 10.6		- 0.68
PRO-6	7.1 ± 3.6	47.6 ± 28.4^b^	39.4 ± 21.3^b^	30.8 ± 16.3	34.6 ± 12.6^b^	28.3 ± 15.4	36.0 ± 25.0	13.7 ± 7.1^b^		- 0.97
TAG (mmol/L)										
CON-2	0.9 ± 0.3	1.2 ± 0.4	1.6 ± 0.6	1.5 ± 0.6	1.6 ± 0.5	1.4 ± 0.6	1.6 ± 0.4	294 ± 133	.026	
CON-6	1.1 ± 0.5	1.1 ± 0.5	1.4 ± 0.5	1.6 ± 0.6	1.6 ± 0.6	1.6 ± 0.5	1.8 ± 0.6	224 ± 139		- 0.52
PRO-2	0.9 ± 0.3	1.1 ± 0.3	1.6 ± 0.6	1.5 ± 0.4	1.7 ± 0.6	1.7 ± 0.5	1.7 ± 0.6	322 ± 91		0.21
PRO-6	1.0 ± 0.5	1.2 ± 0.5	1.6 ± 0.6	1.9 ± 0.8	1.9 ± 0.7	1.9 ± 0.7	2.0 ± 0.7	340 ± 175		0.34
hs-CRP (mg/L)										
CON-2	0.74 ± 1.41	0.79 ± 1.44	-	0.74 ± 1.44	0.69 ± 1.39	-	0.72 ± 1.40	−0.5 ± 90	.17	
CON-6	0.80 ± 1.04	0.81 ± 1.00	-	0.73 ± 0.87	0.66 ± 0.81	-	0.55 ± 0.70	−50 ± 115		- 0.55
PRO-2	0.64 ± 0.86	0.69 ± 0.84	-	0.70 ± 0.82	0.65 ± 0.77	-	0.63 ± 0.71	12 ± 71		0.14
PRO-6	0.12 ± 0.18	0.13 ± 0.21	-	0.15 ± 0.23	0.18 ± 0.24	-	0.17 ± 0.25	19 ± 35		0.22
Systolic BP (mmHg)										
CON-2	120 ± 10	122 ± 10	121 ± 11	120 ± 7	118 ± 8	123 ± 9	122 ± 9	459 ± 3343	.84	
CON-6	118 ± 6	120 ± 6	118 ± 9	116 ± 6	121 ± 8	120 ± 6	120 ± 6	249 ± 2101		−0.06
PRO-2	116 ± 8	123 ± 8	118 ± 11	118 ± 12	120 ± 13	116 ± 6	119 ± 8	1501 ± 4658		0.31
PRO-6	119 ± 8	119 ± 7	118 ± 12	120 ± 9	120 ± 7	118 ± 11	123 ± 9	245 ± 4209		−0.06
Diastolic BP (mmHg)										
CON-2	78 ± 8	73 ± 8	76 ± 9	78 ± 7	71 ± 8	73 ± 8	77 ± 5	−1560 ± 2399	.86	
CON-6	76 ± 6	76 ± 9	74 ± 7	73 ± 6	73 ± 4	76 ± 7	75 ± 6	−614 ± 2272		−0.39
PRO-2	78 ± 9	75 ± 5	74 ± 7	75 ± 9	77 ± 14	72 ± 8	77 ± 8	−1466 ± 3024		−0.04
PRO-6	79 ± 6	77 ± 10	76 ± 6	76 ± 6	76 ± 8	76 ± 8	77 ± 6	−1473 ± 2642		−0.04

All values are mean ± standard deviation. *n* = 10. *iAUC*: Incremental Area under the Curve for 8.5 h period. D: Effect size Cohen’s d, Glass’sΔ correction. Figures with differing superscript letters are significantly different: *p* <0.05 (after Bonferroni correction)

### Postprandial glucose, insulin and triglycerides

For CGMS gluclose, the iAUCs for PRO 2 and PRO 6 were significantly lower than that of both CON 2 and CON 6 (all *P* < 0.05) when compared using least significant differences. The glucose iAUCs for CON-6, PRO-2 and PRO-6 were 11, 66 and 60 % lower respectively, as compared to CON-2. For plasma insulin, the iAUC for PRO 2 and PRO 6 were significantly lower than CON 2 (*P* < 0.05) when compared using least significant differences. The insulin iAUC for CON-6, PRO-2 and PRO-6 were 30, 36 and 52 % lower respectively, compared to CON-2. For plasma TAG, the iAUC for CON-6 was 24 % lower respectively compared to the CON-2 trial. The iAUC for PRO-2 and PRO-6 were slightly higher (10 and 16 % respectively), compared to the CON-2 although these differences were not statistically significant.

### Postprandial hs-CRP, blood pressure

Plasma hs-CRP levels were measured at 0, 1, 4, 5.5 and 8.5-h to obtain an indication of differences in postprandial inflammatory response between the dietary conditions. No statistical significant differences were found. These time points for measurements were selected based on the highest and the lowest fluctuations of the glucose measurements (CGM data) from baseline in order to assess whether postprandial glycaemic variation was associated with changes in hs-CRP levels. There were also no significant differences in systolic or diastolic blood pressure between different dietary conditions.

### Urinary total F2-isoprostanes and creatinine excretion

The mean total urinary excretions of 5-F_2t_-isoprostane, the sum of 15-F_2t_-isoprostane metabolites (2,3-dinor-15-F_2t_-isoprostane and 2,3-dinor-5,6-dihydro-15-F_2t_-isoprostane) and creatinine over the entire 8.5 h postprandial period for each dietary condition is shown in Table 4. Surprisingly, 15-F_2t_-isoprostane (parent compound) being the well-noted biomarker of oxidative stress *in vivo* [40] was found in trace amounts or not detectable in the urine samples of the subjects. Urinary creatinine excretion of 15-F_2t_-isoprostane metabolites during PRO-2 and PRO-6 were significantly higher than CON-2 (*p* < 0.05). There being differences in creatinine excretion between the different dietary conditions, excretion of F_2_-isoprostanes or its metabolites could not be adjusted for creatinine excretion. We did not find any statistical difference between 5-F_2t_-isoprostane between the different dietary conditions, although the sum of 15-F_2t_-isoprostane metabolites were significantly higher in PRO-2 and PRO-6 than CON-2 (*p* < 0.05).

**Table 4:** Total Urinary Excretions of 5-F_2t_-isoprostanes, 15-F_2t_-isoprostanes Metabolites and Creatinine over 8.5 h postprandial period

	CON-2 Mean (SD)	CON-6 Mean (SD)	PRO-2 Mean (SD)	PRO-6 Mean (SD)	Overall p (ANOVA)
5-F_2t_-isoprostanes (μg)	7.8 (13.84)	3.4 (3.96)	2.0 (2.20)	3.2 (4.94)	0.268
Sum of 15-F_2t_-isoprostane metabolites (μg)	2.4 (1.50)	3.2 (2.89)	4.7 (2.53)	3.4 (1.48)	0.059
Creatinine (mmol)	4.2 (1.0)	5.8 (1.8)	8.4 (4.7)	7.6 (2.8)	0.004

## Discussion

To our best knowledge, this is first study to have investigated the combined effects of increasing meal frequency and/or protein composition on range of postprandial cardiometabolic markers in an Asian population. Moreover, in most studies investigating influence of high or low protein with/without changes in meal frequency on postprandial metabolic responses used either supplemental meal replacement powders or liquid diets [17, 41–43], whereas, we have used mixed-composite meals which are better representation of a regular diet. This is also the first study using the continuous glucose monitoring (CGM) to determine the combined effects of increased meal frequency with or without higher protein content to determine PPG response. This having collected glucose data every 5 min over the 8.5 h postprandial period should be more accurate than using capillary/venous glucose samples, which are generally collected at greater sampling intervals. From the previous literature, it appears though that the beneficial effects of increasing meal frequency in improving PPG might be more relevant in those with impaired glucose tolerance (IGT) and diabetes than in healthy individuals [18–21]. In our study we have also demonstrated that increasing meal frequency *per se* did not have a major influence on postprandial glucose control. However, as expected, increasing protein content of the meals (from 10 % of energy to ~40 % of energy) led to significant reductions in PPG by about 60 %, as well as maintained it within 0.5 mmol/l of baseline, irrespective of changes in meal frequency. This was likely to be due to the lower glycemic index (GI) as well as the lower glycemic load (GL) of the high-protein (PRO-2/PRO-6) meals, as compared to the low-protein (CON-2/CON-6) meals. Therefore for the first time, we have been able to show that the GI/GL of the diet seems more important in controlling PPG irrespective of meal frequency. Increased dietary protein intake have been shown to reduce postprandial glycaemic response in several studies both acutely [44, 45] and chronically [32]. On the other hand, postprandial insulinaemic (PPI) responses were lower with increasing meal frequency, independently of meal composition. For example, CON-6 dietary condition had approximately 30 % lower postprandial iAUC of insulin compared with CON-2 dietary condition, whereas, PRO-6 dietary condition had 24 % lower iAUC compared with PRO-2 and these may be physiologically relevant. Similar observations of lower postprandial insulin response with increasing meal frequency have also been observed by several other investigators [16, 18, 21]. Moreover, the combined effects of higher protein intake with increased meal frequency gave rise to the lowest iAUC for postprandial insulin compared with the three other dietary conditions. While it is well known that dietary protein is insulinogenic [46], in this study we have shown that when the same quantity and quality of protein in diet is separated into smaller boluses through increasing meal frequency (PRO-6), the total PPI response was much lower than having the same meal as larger boluses (PRO-2). At the same time, we did not find any major difference in the postprandial glycaemic response between PRO-2 and PRO-6 dietary conditions, which may indirectly indicate that the insulin sensitivity in conditions of high protein and high meal frequency (PRO-6) might be greater than PRO-2. Although insulin sensitivity was not directly tested in our study, similar observations were made in other longer term studies, where a moderate intake of 20–25 % protein had given rise to better insulin sensitivity than a higher dietary protein (25–30 % protein as energy) intake [47]. It should also be noted that different protein types (e.g., whey, casein, soy) can give rise to variability in postprandial insulinaemic response [45] and this has to be taken into consideration before arriving at any meaningful conclusion regarding dietary protein content and insulin sensitivity. Postprandial hypertriglyceridaemia is associated with increased CVD and type 2 diabetes risk [48, 49]. Therefore the 24 % lower mean iAUC of postprandial triglyceridaemia (TAG) with increasing meal frequency in the low protein-high carbohydrate (CON-6) condition as compared with CON-2 condition is likely to be physiologically relevant, even though this was not statistically significant due to the large inter-individual variability.

Given that the postprandial state is also associated with an increased oxidative stress and/or inflammation [48, 50], we investigated whether high and low protein diets, in combination with high and low meal frequency patterns can modify markers of postprandial oxidative stress or inflammation. We did not find any significant influence of altering meal frequency *per se* on these parameters and these findings are similar to other studies where no differences in oxidative stress markers upon changing meal frequency were observed [51]. However, we found that while the excretion 15-F_2t_-isoprostane metabolites (2,3-dinor-15-F_2t_-isoprostane and 2,3-dinor-5,6-dihydro-15-F_2t_-isoprostane) increased with higher protein intake (PRO-2 and PRO-6 combined vs CON-2 and CON-6 combined, *p* < 0.05), the excretion of 5-F_2t_-isoprostane was in fact lower with higher protein intakes (74 and 59 % lower in PRO-2 and PRO-6 diets respectively as compared to the CON-2 diet), although none of these differences were statistically significant. It should be noted that the urine samples of the subjects in this study had only trace concentrations of 15-F_2t_-isoprostane compared to 5-F_2t_-isoprostane; similar findings were reported in other studies [52]. This, in addition to the increased renal clearance, as evidenced by greater creatinine excretion with higher protein diets, explained the higher excretion of 15-F_2t_-isoprostane metabolites in PRO-2 and PRO-6 diets compared with the low protein (CON-2 and CON-6) diets. We have also compared the ratios of F_2_-isoprostane to creatinine excretion for the separate of F_2_-isoprostane species between the different dietary conditions, although there were no statistical differences. Thus, increased excretion of 15-F_2t_-isoprostane metabolites in this study with increased protein intake may not necessarily indicate increased oxidative stress with the higher protein diets, particularly given that the 5-F_2t_-isoprostane excretion was much lower and that 5-F_2t_-isoprostane could well be better marker of *in vivo* oxidative stress under some circumstances [53]. The higher creatinine excretion with increasing protein intake (PRO-2 and PRO-6 vs CON-2 and CON-6) was similar to other observations which could be due to a higher glomerular filtration rate [54, 55]. However, in healthy volunteers without any kidney disorder, there is no evidence to show that having a high protein diet is detrimental to kidney health [56, 57]. While there are some indications that dietary carbohydrate load is associated with increased CRP levels [58], we did not find any effect of varying meal composition in our study on postprandial hsCRP levels. While postprandial hsCRP levels was unaltered with the acute dietary modulations, it remains to be investigated how similar dietary changes may affect hsCRP in the longer term. Finally, one of the major limitations of the study was that the postprandial responses were tested after dietary manipulation for one day only. How longer term dietary manipulations will affect these responses remain to be investigated.

## Conclusions

In contrast to previous studies that have used meal replacement liquid based formulas to undertake similar investigations, we have for the first time demonstrated that it is feasible to use solid composite mixed meals to study combined effect of high protein content and increased meal frequency on markers of postprandial cardiometabolic risk. As expected, we found that increasing protein content of meals decreased PPG response. Contrary to the commonly held view that increasing meal frequency attenuates postprandial glycaemia, our results seem to indicate that the overall protein content and the consequent glycemic index and/or glycemic load of the meals may be more important than meal frequency alone. There were some indications that increasing meal frequency may benefit postprandial insulinemia. Increasing meal frequency and/or protein composition did not affect the other postprandial markers of cardiometabolic risk measured in this study. However, these remain to be tested in larger populations using longer term trials. The findings of this study will have important implications in Asians who already consume diets rich in carbohydrates with high glycaemic index. In order to improve their cardiometabolic risk, Asians may increase the protein content of the meal, irrespective of any need to change their meal frequency.

## Abbreviations

ANOVA: Analysis of Variance
A*STAR: Agency for Science Technology and Research, Singapore
CGM: Continuous Glucose Monitor
CON-2: 2 large Low-Protein iso-caloric meals
CON-6: 6 small Low-Protein iso-caloric meals
CMD: Cardiometabolic Disease
CNRC: Clinical Nutrition Research Centre
CVD: Cardiovascular Disease
hs-CRP: High-sensitivity C-reactive Protein
iAUC: incremental area under the curve
PPG: Postprandial glucose
PRO-2: 2 large High-protein iso-caloric meals
PRO-6: 6 small High-protein iso-caloric meals
SD: Standard Deviation
TAG: Triacylglycerol

## References

[1] Ceriello A (2000). The post prandial state and cardiovascular disease: relevance to diabetes mellitus. Diabetes Metab. Res. Rev..

[2] O’Keefe JH, Bell DS (2007). Postprandial hyperglycemia/hyperlipidemia (postprandial dysmetabolism) is a cardiovascular risk factor. Am J Cardiol.

[3] Greenfield JR (2007). Effect of postprandial insulinemia and insulin resistance on measurement of arterial stiffness (augmentation index). Int J Cardiol.

[4] Peddie MC, Rehrer NJ, Perry TL (2012). Physical activity and postprandial lipidemia: are energy expenditure and lipoprotein lipase activity the real modulators of the positive effect?. Prog Lipid Res.

[5] He W (2013). Greater abdominal fat accumulation is associated with higher metabolic risk in Chinese than in white people: an ethnicity study. PLoS One.

[6] Dickinson S (2002). Postprandial hyperglycemia and insulin sensitivity differ among lean young adults of different ethnicities. J. Nutr.

[7] Cruz ML, Evans K, Frayn KN (2001). Postprandial lipid metabolism and insulin sensitivity in young Northern Europeans, South Asians and Latin Americans in the UK. Atherosclerosis.

[8] Razak F (2005). Ethnic differences in the relationships between obesity and glucose-metabolic abnormalities: a cross-sectional population-based study. Int J Obes.

[9] Choi SE (2013). Gender and ethnic differences in the prevalence of type 2 diabetes among Asian subgroups in California. J Diabetes Complicat.

[10] Zaninotto P, Mindell J, Hirani V (2007). Prevalence of cardiovascular risk factors among ethnic groups: results from the Health Surveys for England. Atherosclerosis.

[11] Bakker LE (2014). A 5-day high-fat, high-calorie diet impairs insulin sensitivity in healthy, young South Asian men but not in Caucasian men. Diabetes.

[12] FAíBRY P, Tepperman J (1970). Meal frequency—a possible factor in human pathology. Am J Clin Nutr.

[13] La Bounty PM (2011). International Society of Sports Nutrition position stand: meal frequency. J Int Soc Sports Nutr.

[14] Jenkins DJ (1989). Nibbling versus gorging: metabolic advantages of increased meal frequency. N Engl J Med.

[15] Jones PJ, Leitch CA, Pederson RA (1993). Meal-frequency effects on plasma hormone concentrations and cholesterol synthesis in humans. Am J Clin Nutr.

[16] Munsters MJ, Saris WH (2012). Effects of meal frequency on metabolic profiles and substrate partitioning in lean healthy males. PLoS One.

[17] Holmstrup ME (2010). Effect of meal frequency on glucose and insulin excursions over the course of a day. e-SPEN, Eur e-J Clin Nutr Metab.

[18] Bertelsen J (1993). Effect of meal frequency on blood glucose, insulin, and free fatty acids in NIDDM subjects. Diabetes Care.

[19] Jenkins DJ (1992). Metabolic advantages of spreading the nutrient load: effects of increased meal frequency in non-insulin-dependent diabetes. Am J Clin Nutr.

[20] Allirot X (2014). Effects of a breakfast spread out over time on the food intake at lunch and the hormonal responses in obese men. Physiol Behav.

[21] Solomon TP (2008). The effect of feeding frequency on insulin and ghrelin responses in human subjects. Bri J Nutr.

[22] Abeywardena MY (2003). Dietary fats, carbohydrates and vascular disease: Sri Lankan perspectives. Atherosclerosis.

[23] Feinman RD (2015). Dietary carbohydrate restriction as the first approach in diabetes management: Critical review and evidence base. Nutrition.

[24] Volek JS (2009). Carbohydrate restriction has a more favorable impact on the metabolic syndrome than a low fat diet. Lipids.

[25] Volek JS (2008). Dietary carbohydrate restriction induces a unique metabolic state positively affecting atherogenic dyslipidemia, fatty acid partitioning, and metabolic syndrome. Prog Lipid Res.

[26] Arciero PJ (2006). Increased dietary protein and combined high intensity aerobic and resistance exercise improves body fat distribution and cardiovascular risk factors. Int J Sports Nutr Exer Metab.

[27] Arciero PJ (2008). Moderate protein intake improves total and regional body composition and insulin sensitivity in overweight adults. Metabolism.

[28] Krieger JW (2006). Effects of variation in protein and carbohydrate intake on body mass and composition during energy restriction: a meta-regression. Am J Clin Nutr.

[29] Layman DK (2003). Increased dietary protein modifies glucose and insulin homeostasis in adult women during weight loss. J Nutr.

[30] Claessens M (2007). The thermogenic and metabolic effects of protein hydrolysate with or without a carbohydrate load in healthy male subjects. Metabolism.

[31] Heer M, Egert S (2015). Nutrients other than carbohydrates: their effects on glucose homeostasis in humans. Diabetes Metab. Res. Rev..

[32] Farnsworth E (2003). Effect of a high-protein, energy-restricted diet on body composition, glycemic control, and lipid concentrations in overweight and obese hyperinsulinemic men and women. Am J Clin Nutr.

[33] Singapore HPB (2010). Report of the National Nutrition Survey 2010 Singapore.

[34] Hätönen KA (2011). Protein and fat modify the glycaemic and insulinaemic responses to a mashed potato-based meal. Br J Nutr.

[35] Chen Y-J (2010). Glycemic index and glycemic load of selected Chinese traditional foods. World J Gastroenterol.

[36] Atkinson FS, Foster-Powell K, Brand-Miller JC (2008). International tables of glycemic index and glycemic load values: 2008. Diabetes Care.

[37] Lee YY (2015). Prenatal exposure to the contaminant perfluorooctane sulfonate elevates lipid peroxidation during mouse fetal development but not in the pregnant dam. Free Radic Res.

[38] Chung MLS, Lee KYE, Lee C-YJ (2013). Profiling of oxidized lipid products of marine fish under acute oxidative stress. Food Chem Toxicol.

[39] Pruessner JC (2003). Two formulas for computation of the area under the curve represent measures of total hormone concentration versus time-dependent change. Psychoneuroendocrinology.

[40] Morrow JD (1990). A series of prostaglandin F2-like compounds are produced in vivo in humans by a non-cyclooxygenase, free radical-catalyzed mechanism. Proc Natl Acad Sci U S A.

[41] Jones PJ, Leitch CA, Pederson RA. Meal-frequency effects on plasma hormone concentrations and cholesterol synthesis in humans. The American journal of clinical nutrition. 1993;57(6):868-74. 10.1093/ajcn/57.6.868 8503355

[42] Heden TD (2013). Liquid meal composition, postprandial satiety hormones, and perceived appetite and satiety in obese women during acute caloric restriction. Eur J Endocrinol.

[43] Kanaley JA (2014). Alteration of postprandial glucose and insulin concentrations with meal frequency and composition. Br J Nutr.

[44] El Khoury D (2014). Increasing the protein to carbohydrate ratio in yogurts consumed as a snack reduces post-consumption glycemia independent of insulin. Clin Nutr.

[45] Acheson KJ (2011). Protein choices targeting thermogenesis and metabolism. Am J Clin Nutr.

[46] Holt S, Miller J, Petocz P (1997). An insulin index of foods: the insulin demand generated by 1000-kJ portions of common foods. Am J Clin Nutr.

[47] Weickert MO (2011). Effects of supplemented isoenergetic diets differing in cereal fiber and protein content on insulin sensitivity in overweight humans. The Am J Clin Nutr.

[48] Anderson RA (2001). The relationships between post-prandial lipaemia, endothelial function and oxidative stress in healthy individuals and patients with type 2 diabetes. Atherosclerosis.

[49] Groot P (1991). Postprandial lipoprotein metabolism in normolipidemic men with and without coronary artery disease. Arterioscler Thromb Vasc Biol.

[50] O’Keefe JH, Gheewala NM, O’Keefe JO (2008). Dietary strategies for improving post-prandial glucose, lipids, inflammation, and cardiovascular health. J Am Coll Cardiol.

[51] Heden TD (2013). Meal frequency differentially alters postprandial triacylglycerol and insulin concentrations in obese women. Obesity (Silver Spring).

[52] Li H (1999). Quantitative high performance liquid chromatography/tandem mass spectrometric analysis of the four classes of F2-isoprostanes in human urine. Proc Natl Acad Sci.

[53] Halliwell B, Lee CYJ (2009). Using Isoprostanes as Biomarkers of Oxidative Stress: Some Rarely Considered Issues. Antioxid. Redox Signal..

[54] Lew S, Bosch JP (1991). Effect of diet on creatinine clearance and excretion in young and elderly healthy subjects and in patients with renal disease. J Am Soc Nephrol.

[55] Denis W (1917). A note on the diurnal variations in creatine excretion. J Biol Chem.

[56] Brenner B, Meyer T, Hostetter TH (1982). Dietary protein intake and the progressive nature of kidney disease: the role of hemodynamically mediated glomerular injury in the pathogenesis of progressive glomerular sclerosis in aging, renal ablation, and intrinsic renal disease. N Engl J Med.

[57] Brändle E, Sieberth H, Hautmann R (1996). Effect of chronic dietary protein intake on the renal function in healthy subjects. Eur J Clin Nutr.

[58] Clifton PM (2003). Diet and C-reactive protein. Curr. Atheroscler. Rep..

